# Direct Integration of Strained‐Pt Catalysts into Proton‐Exchange‐Membrane Fuel Cells with Atomic Layer Deposition

**DOI:** 10.1002/adma.202007885

**Published:** 2021-06-10

**Authors:** Shicheng Xu, Zhaoxuan Wang, Sam Dull, Yunzhi Liu, Dong Un Lee, Juan S. Lezama Pacheco, Marat Orazov, Per Erik Vullum, Anup Lal Dadlani, Olga Vinogradova, Peter Schindler, Qizhan Tam, Thomas D. Schladt, Jonathan E. Mueller, Sebastian Kirsch, Gerold Huebner, Drew Higgins, Jan Torgersen, Venkatasubramanian Viswanathan, Thomas Francisco Jaramillo, Fritz B. Prinz

**Affiliations:** ^1^ Department of Mechanical Engineering Stanford University Stanford CA 94305 USA; ^2^ Department of Material Science and Engineering Stanford University Stanford CA 94305 USA; ^3^ Department of Chemical Engineering Stanford University Stanford CA 94305 USA; ^4^ Department of Earth System Science Stanford University Stanford CA 94305 USA; ^5^ SINTEF Trondheim 7465 Norway; ^6^ Department of Mechanical and Industrial Engineering Norwegian University of Science and Technology Trondheim 7491 Norway; ^7^ Chemical Engineering Carnegie Mellon University Pittsburgh PA 15213 USA; ^8^ Volkswagen Group Research 38436 Wolfsburg Germany; ^9^ Department of Chemical Engineering McMaster University Hamilton ON L8S 4L7 Canada; ^10^ Mechanical Engineering Carnegie Mellon University Pittsburgh PA 15213 USA

**Keywords:** fuel cells, lattice strain, membrane electrode assembly, oxygen reduction reaction, rotating disk electrodes

## Abstract

The design and fabrication of lattice‐strained platinum catalysts achieved by removing a soluble core from a platinum shell synthesized via atomic layer deposition, is reported. The remarkable catalytic performance for the oxygen reduction reaction (ORR), measured in both half‐cell and full‐cell configurations, is attributed to the observed lattice strain. By further optimizing the nanoparticle geometry and ionomer/carbon interactions, mass activity close to 0.8 A mg_Pt_^−1^ @0.9 V iR‐free is achievable in the membrane electrode assembly. Nevertheless, active catalysts with high ORR activity do not necessarily lead to high performance in the high‐current‐density (HCD) region. More attention shall be directed toward HCD performance for enabling high‐power‐density hydrogen fuel cells.

## Graphical abstract

Designer electrocatalysts can be deposited via atomic layer deposition onto support structures for performance evaluation under various platforms. In this example, strained platinum catalysts are formed by dissolving cobalt oxide from the Pt/CoO*_x_* core–shell structure. Performance enhancement seen on the rotating disk electrode is validated in the membrane electrode assembly.

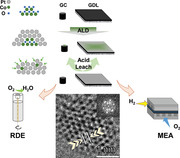

## 1 Introduction

Catalysts for the oxygen reduction reaction (ORR) have been studied extensively^[^ ^1^, ^2^, ^3^, ^4^, ^5^, ^6^, ^7^ ^]^ as fuel cells begin to emerge as one of the major clean and sustainable energy conversion technologies. Among various strategies to improve the intrinsic catalytic activity of Pt‐based catalysts, strain engineering has shown promises in tuning the surface reactivity by changing the atomic spacing of Pt.^[^ ^8^, ^9^, ^10^ ^]^ Strain alters the d‐band center of catalytic materials,^[^ ^11^, ^12^, ^13^ ^]^ which plays a crucial role in the energetics of adsorption of oxygenic species to the catalytic surfaces.^[^ ^13^, ^14^, ^15^ ^]^ A common method for introducing surface strain involves alloying Pt with transition metals.^[^ ^16^, ^17^, ^18^ ^]^ A variety of nanostructures including nanoparticle,^[^ ^19^, ^20^, ^21^, ^22^ ^]^ nanowire,^[^ ^3^, ^23^, ^24^ ^]^ nanoframes,^[^ ^5^, ^25^ ^]^ and nanocages^[^ ^7^, ^26^ ^]^ have been implemented on Pt alloy catalysts with high ORR activity. In addition to chemically induced global strain in alloys, local structural strain has also been proven useful for ORR activity enhancement.^[^ ^27^ ^]^ Core–shell catalysts composed of a Pt shell and Pt–M alloy core have become a promising candidate,^[^ ^6^, ^18^, ^21^, ^28^, ^29^, ^30^, ^31^ ^]^ where fine‐tuned shell dimensions^[^ ^18^, ^32^ ^]^ and core composition^[^ ^6^, ^30^ ^]^ can lead to further optimized activity.^[^ ^33^ ^]^ With even more transition metal removed, hollow structures with greater portion of Pt atoms exposed for catalytic reactions can be achieved via acid treatment and galvanic replacements.^[^ ^34^, ^35^, ^36^ ^]^

Despite high activities reported in half‐cell setups such as in a rotating disk electrode (RDE), only a few of these catalysts have demonstrated an enhanced catalytic performance at the membrane electrode assembly (MEA) level that is more relevant to fuel cell performances. Moreover, there has been a reported mismatch between RDE and MEA performance,^[^ ^37^, ^38^, ^39^ ^]^ where low correlation is found.^[^ ^40^ ^]^ These discrepancies can be attributed to multiple differences between RDE and MEA. A non‐adsorbing electrolyte such as perchloric acid is normally applied in an RDE setup, whereas sulfonated species, which more readily adsorb to Pt catalysts, are applied in the MEA. Ionomer‐free^[^ ^41^, ^42^ ^]^ studies are possible with RDE while ionomers are indispensable for MEA setups.^[^ ^43^, ^44^, ^45^ ^]^ In addition, the activity of oxygen in the two systems is drastically different in that catalysts are exposed to more oxygen in the MEA which influences formation kinetics of Pt oxides.^[^ ^46^, ^47^, ^48^, ^49^, ^50^ ^]^ Moreover, the interference of the counter electrodes^[^ ^51^, ^52^ ^]^ may also contribute to the difference in measured activity. In short, it is possible to quantify the intrinsic catalytic activity of the designed catalytic structure with an RDE; however, the design of a catalytic system in the MEA composed of catalyst, support, ionomer, and electrolyte is a grand challenge with additional degrees of complexity.

In this work, we report Pt catalysts developed with atomic layer deposition (ALD) techniques^[^ ^53^ ^]^ evaluated under both RDE and MEA. Leveraging the layer‐by‐layer deposition of ALD, we fabricated core–shell structures with Pt as the shell and soluble metal oxide as the core, which would be otherwise challenging to synthesize using solution‐based synthetic approaches. After dissolving the ALD oxide core, we were able to introduce strain directly into the Pt catalysts and study the correlated enhancement in the catalytic activity in both testing setups. By properly adjusting the ionomer and catalyst structures, the mass activity of the strained Pt catalysts can be made to approach 0.8 A mg_Pt_^−1^ (@0.9 V iR‐free). The streamlined device‐level research process for catalyst development using ALD is detailed below.

## 2 Results and Discussion

### 2.1 Synthesizing Designed Catalyst with Atomic Layer Deposition

**Figure 1:**
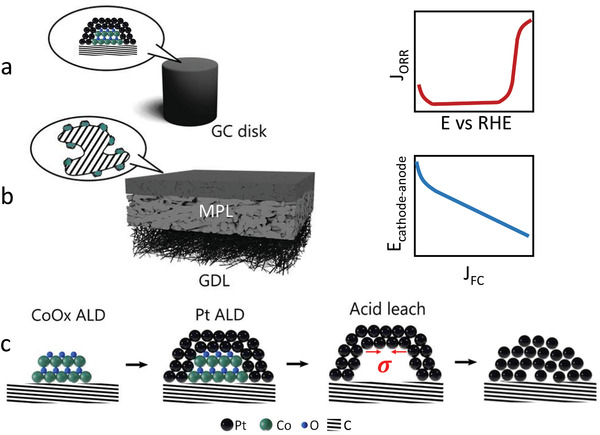
a–c) Catalyst functionalization and evaluation schematics in: a) RDE and b) MEA, and c) the catalyst design and synthesis process.

Pt catalysts were fabricated via direct ALD deposition onto glassy carbon disks and carbon‐loaded gas diffusion layers (GDL), as laid out in **Figure** **1**a,b. By anchoring Pt nanoparticles on the glassy carbon surface, no binders are necessary. Therefore, the RDE tests allow ORR activity evaluation without the interference of ionomer. In the MEA tests, the catalyst, catalyst support, ionomer dosage, and incorporation methods can be varied systematically to study their effects on catalytic performance. It not only allows more degrees of freedom in electrode optimization, this method offers a direct MEA level performance quantification on catalyst materials as‐synthesized, suitable for designing and developing adventurous catalyst structures. Although this report focuses primarily on catalyst development, influence of the carbon support on activity will be briefly showcased at the end. The use of poly(tetrafluoroethylene) (PTFE)‐containing microporous layer allows limited catalyst deposition in the catalyst layer, because PTFE serves as an inhibitor for Pt ALD, which helps confining Pt in the catalyst layer. The cathode gas diffusion electrode (GDE) is fabricated sequentially with ALD on carbon‐loaded GDL, acid‐leaching post‐treatment, and ionomer impregnation. It is then pressed against a membrane with an anode GDE to complete the entire MEA. ALD recipes and post‐treatment conditions were varied on both RDE and MEA for understanding the activity limit of the synthesized catalysts.

Strained Pt catalysts were implemented with sequential ALD deposition of template cobalt oxide and Pt, followed by almost completely dissolving of the template with acid. Both the Pt and CoO*_x_* ALD cycles used in the study are within the nucleation phase of film deposition, leading to nanoparticle formation. Cobalt is covered by Pt after the sequential ALD deposition as confirmed with the XPS depth profile (Figure S1, Supporting Information). After the cobalt oxide template is removed with acid leaching, it is expected that two categories of catalytic structures form. For structures with the platinum overlayer completely covering the template, removal of the template will subject the overlayer to a pressure difference, as shown by Figure 1c. It can either form a hollow structure or collapse. Both scenarios will leave behind a compressive strain that is positively correlated to the size of the template and negatively correlated to the thickness of the platinum overlayer film. For the structures with platinum covering part of the template, platinum particles that have anchoring to the catalyst support will end up exposing more catalytic active surfaces. The platinum particles that have weak bonding to the catalyst support will be lifted off. Due to the high surface energy of Pt, these particles are likely to redeposit on the other part of the support materials. This is especially applicable to the GDEs made with this method, where acid leaching can remove the majority of the cobalt oxide without noticeably changing the mass loading of Pt.

### 2.2 Comparing Rotating Disk Electrode and Membrane Electrode Assembly

**Figure 2:**
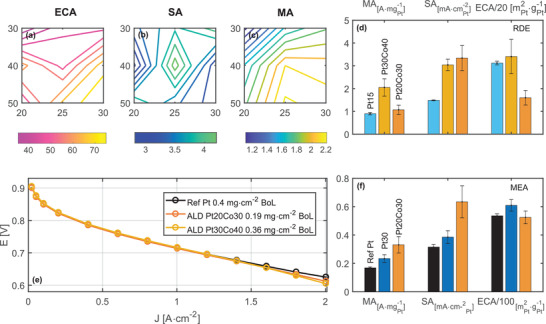
Electrochemical performance of strained ALD Pt catalysts. a) Electrochemically active area, b) specific activity, and c) mass activity @0.9 V versus RHE of strained Pt/CoO*_x_* catalysts deposited by varied Pt and CoO*_x_* ALD cycles. d) Comparison of these performance metrics of Pt30Co40, Pt20Co30, and Pt15 catalysts in the RDE. e) Fuel cell performance of 5 cm^2^ MEAs with Pt20Co30 (0.19 mg cm^−2^), Pt30Co40 (0.36 mg cm^−2^), and Pt (0.4 mg cm^−2^) cathodes under 80 °C, 100 % RH, 150 kPa_abs_, and 0.5/5 L min^−1^ H_2_/air flow are compared by polarization curves taken with sequences specified by the US DOE protocol. f) Comparison of the performance metrics of the two ALD catalysts versus a reference Pt MEA @0.9 V iR‐free. The average values and error bars were obtained as the standard deviation of measurements from five replicate samples.

Catalysts were deposited with ALD onto glassy carbon electrodes and carbon‐loaded GDL, for catalytic performance evaluation under RDE and MEA, respectively. Acid‐leaching conditions were tested to achieve reproducible results. When no pretreatment is applied in the RDE measurement, it is equivalent to pretreating the as‐deposited electrode with strong acid (electrolyte pH = 1). Cobalt oxide quickly dissolves, which leads to drastic liftoff of Pt overlayers, causing a loss in Pt mass and kinetic current as well as large variation in results. Therefore, pH = 4 (Figure S2, Supporting Information) was chosen for preleaching in the RDE studies. It is well acknowledged that Pt‐bimetallic catalyst systems can undergo structural change and non‐previous metal dissolution under electrochemical testing condition.^[^ ^54^, ^55^ ^]^ Therefore, multiple voltammetry scans were taken until convergence arrives to make sure the electrochemically active surfaces are stabilized (Figure S3, Supporting Information). The ALD cycles of Pt and CoO*_x_* deposition were varied to understand the interaction between the Pt overlayer and the CoO*_x_* template. The resulting structures were compared at a Pt mass loading range of 1.4–2.5 ug cm^−2^ with a fixed RDE test protocol. **Figure** **2**a shows the electrochemically active area (ECA) as a function of Pt and CoO*_x_* ALD cycles. The growth rate of Pt ALD is noticeably slower on CoO*_x_* layers. As a result, increased CoO*_x_* ALD cycles lead to higher ECA, which corresponds to smaller‐sized nanoparticles. The increased ECA with higher Pt ALD cycles cannot be explained by growth of Pt nanoparticles. Rather, it implies a stronger Pt/CoO*_x_* interaction during leaching when the coverage of Pt over CoO*_x_* increases with high Pt ALD cycles. Overall, the ECA contour suggests that larger Pt nanoparticles form with high Pt and low CoO*_x_* cycle numbers, and smaller nanoparticles form with high Pt and high CoO*_x_* cycle numbers. The former converges to large Pt nanoparticles with high specific activity closer to that of polycrystalline Pt. For any strain remaining in the leached structure, its magnitude should scale with the ratio of thicknesses between CoO*_x_* and Pt. With an increased ratio between cycle numbers of CoO*_x_* and Pt, strain effects become more pronounced. Nevertheless, low Pt ALD cycle numbers also lead to smaller sized Pt particles with lower specific activity. Therefore, an optimal specific activity is reached with moderate Pt and CoO*_x_* cycles, as shown by Figure 2b. The key performance metric, mass activity (Figure 2c), is the product of specific activity and ECA, which is favored by high Pt and high CoO*_x_* cycle numbers. Figure 2d compares two ALD catalysts with similar ECA. The Pt30Co40 (30 Pt and 40 CoO*_x_* ALD cycles) outperforms Pt15 (15 Pt ALD cycle) with more than double the mass activity, mostly attributed to the improvement in specific activity. The optimal mass activity is achieved on Pt25Co30 at around 2.1 A mg_Pt_^−1^, and most Pt/CoO*_x_* catalyst achieved specific activity at least twice that of Pt (Table S1, Supporting Information). The performance improvement mainly attributes to the Pt component as minute amount of cobalt was found after electrochemical testing (Figure S4, Supporting Information). ICP‐MS detected Co/Pt atomic ratio drops from 30% to 40% in as‐deposited samples to <3% after the RDE tests.

The same optimization was applied in the MEA studies where Pt and CoO*_x_* ALD cycles were varied, and pH = 3.5 was chosen as the preleaching condition. Similar to the findings on RDE (Figure S4, Supporting Information), there is a minute amount (<3 atomic% to that of Pt) of cobalt left in the catalysts. The ALD growth rates are faster on the carbon‐loaded GDEs than on glassy carbon due to shorter nucleation delays on amorphous carbon structures. The GDE with Pt30Co40 recipe resulted in larger particles than those on glassy carbons. In addition to the differences in the RDE and MEA ORR conditions mentioned earlier, the optimal ALD recipe for MEA differs from that for the RDE, identified here as Pt20Co30 (20 Pt and 30 CoO*_x_* ALD cycles). Shown in Figure 2d, the same recipe on glassy carbon disks leads to smaller ECA compared to those particles grown on the GDE. With twice the specific activity of a state‐of‐the‐art Pt reference cathode catalyst, Pt20Co30 gives a similar polarization curve with less than half the Pt loading, as shown in Figure 2e. A reference sample without CoO*_x_* but only 30 cycles of Pt ALD (Pt30) was chosen to compare with respect to its similar ECA. Despite having a higher MA than the reference Pt, Pt30 has a SA significantly lower than that of the Pt20Co30. Overall, the benefits of specific activity improvement shown in RDE are successfully translated to MEA, but with a value approximately five times lower. As surveyed by Yarlagadda et al.,^[^ ^40^ ^]^ the ratio of activity between that in the MEA (often measured and compared at 80 °C) and that in the RDE (close to room temperature) spans over a wide range from one twentieth to almost unity. Setting aside the measurement protocol differences and the variations among electrode designs, catalytic systems entailed with different activation energies respond to temperature differently. Specifically, catalysts with higher intrinsic activities benefit less from increases in temperature. For highly active systems, the thermodynamic loss in reversible cell potential at high temperatures can even outweigh the kinetic enhancement. Therefore, there is no universal ratio that can correlate RDE and MEA results. The degree of enhancement translation by the Pt/CoO*_x_* system implies an activation energy barrier reduction of ≈5 kJ mol^−1^ compared to that of Pt.

### 2.3 Probing Strained Catalysts

**Figure 3:**
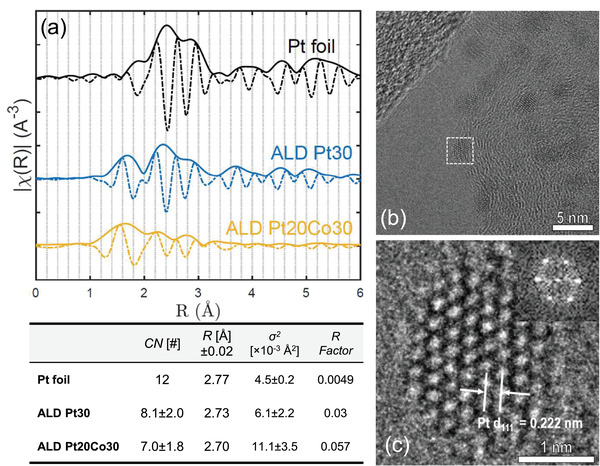
Strain analysis of ALD Pt catalysts. a) Fourier transform of the EXAFS spectra of the Pt L_3_ edge Pt foil, ALD Pt30, and ALD Pt20Co30 with amplitude (solid) and real part (dashed). The fitting results summarized by the table below was obtained by considering only the first Pt shell, using data range of 2.05 < *R* < 3.15 Å and Δ*k* = 3–11.5 Å^−1^, with So^2^ fixed at 0.83 (obtained from a Pt foil fit). b,c) HRTEM images of the ALD Pt20Co30 sample with (111) plane spacing (b), measured on a particle viewed from a zone axis of [110] (c).

X‐ray absorption spectroscopy (XAS) and transmission electron microscopic (TEM) studies were conducted to understand the structures of selected catalysts. These two complementary techniques provide global and local views, respectively. The XAS on the Pt L_3_ edge was examined and the Fourier transform amplitude of extended X‐ray absorption fine structure (EXAFS) spectra of 30 cycles of Pt ALD (Pt30) and the 20 cycles of Pt ALD after 30 cycles of CoO*_x_* ALD followed by acid leaching (Pt20Co30) are compared in **Figure** **3**a. Compared to Pt foil, both ALD samples show the existence of Pt—O. This is manifested by the Pt—O contribution peak around 1.6 Å and the decreased amplitude of the Pt—Pt nearest neighbor contributions at around 2.2 Å.^[^ ^56^ ^]^ XANES also suggests Pt20Co30 is less metallic than Pt30 and the reference Pt foil (Figure S5a, Supporting Information). The TEM observations do not show crystalline Pt oxide structures, which implies that the Pt—O bonds correspond to amorphous oxides formed at the catalyst surfaces. The nanoparticles are subject to surface oxidation once exposed to the ambient environment.^[^ ^57^ ^]^ For Pt20Co30, the Pt—O is more significant which is attributed additionally to Pt growth on CoO*_x_*. After acid leaching, Pt can bond to oxygen residuals from CoO*_x_*, either from the Pt/CoO*_x_* interface or from the CoO*_x_*/C interface. There is a noticeable downshift at the first‐shell Pt—Pt peak around 2.2–2.4 Å, when comparing Pt20Co30 to both Pt30 and the reference Pt, which can be attributed either to a compressive strain in the Pt—Pt lattice or to an increased interference of the Pt—O species with a shorter bond distance. The first‐shell fitting^[^ ^58^ ^]^ results based on the Pt lattice compression assumption is compiled in the table below Figure 3a. The fitting was first performed on the reference Pt foil data where the So^2^ was fixed at 0.83. The first‐shell Pt—Pt distance was obtained as 2.77 Å, which is in good agreement with theoretical value. The fitting results of ALD samples both show decreased Pt—Pt bond distances. The lower coordination numbers of ALD samples are in agreement with Pt residing in nanoparticles with less coordinated edge atoms. A 2.5% decrease in Pt—Pt distance for the Pt20Co30 sample compared to the reference sample suggests a significant compressive strain. Taking the latter assumption (results shown in Table S2, Supporting Information), the Pt—Pt bonds in the assumed Pt—O species of Pt20Co30 is more than 5% shorter than that in the Pt30 sample. With either assumption, the shift in the spectra implies a compressive strain in the Pt lattices of either the nanoparticle or of the surface oxide. The TEM results incline to accept the first hypothesis. As shown in the high‐resolution TEM image and its FFT result from Figure 3c, the Pt nanoparticle was viewed from a zone axis of [110]. Based on the measurement along its (111) crystal planes, the d‐spacing of (111) planes is 2.22 Å which indicates a compressive strain in the nanoparticle by 2% (compared to the theoretical value of 2.26 Å, also compared to the measurement from a commercial Pt/C sample as shown by Figure S6, Supporting Information). X‐ray diffraction (XRD) patterns of the Pt ALD, Pt20Co30, and Pt_3_Co alloy were compared in Figure S10, Supporting Information. The strain in the Pt_3_Co alloy can be observed by the peak shift of Pt (111) from 39.7° for Pt ALD to 41.0° for Pt_3_Co. Pt20Co30 also contains such features as a result of strain in addition to those from Pt ALD.

Pt—Pt bond distances had been correlated with the size of the nanoparticles, where lowered coordination numbers in smaller particles result in shortened Pt—Pt distances. Such size‐related Pt strain does not lead to improvement of ORR performance as lower specific activities are often observed with smaller particles. In contrast, the strained Pt20Co30 catalyst has a significantly higher specific activity, and such an activity improvement may be attributed to either the remaining minute amount of Co or the extra strain that have been observed. The Co ligand effects require a considerable amount of Co residing in the near surface atomic layers of the catalyst nanoparticle. According to the XAS measurement on the Co K edge (Figure S7, Supporting Information), the majority of Co was found to be bonded to O or C after MEA testing. The XANES spectra (Figure S5b, Supporting Information) also suggest an absence of Pt—Co alloying, compared to the Pt_3_Co alloy where a significant down shift of features can be observed. A negligible amount of Co—M bonding remained where M cannot be differentiated from Pt and Co. STEM‐EELS was attempted but did not show a Co signal due to the low atomic percentage of Co. In addition, the catalytic activity is not positively correlated to the Co content in the MEA (Figure S8, Supporting Information). Therefore, the existence of Co does not appear to contribute to the activity improvement.

### 2.4 Mass Activity Optimization with Passivation Gas Incorporated Atomic Layer Deposition

**Figure 4:**
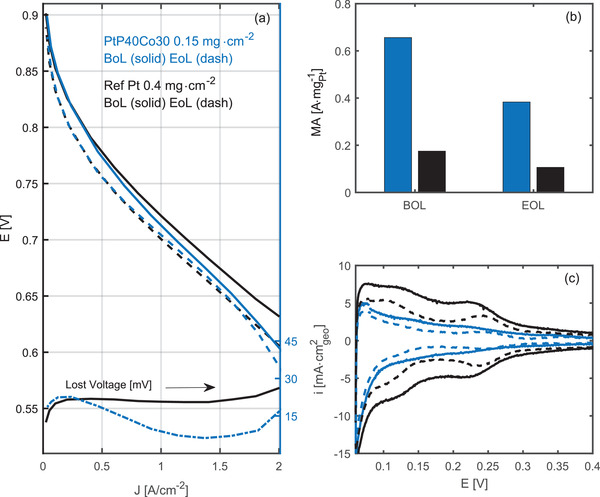
MEA performance of low‐loaded strained Pt catalyst. a) Fuel cell performance of 5 cm^2^ MEAs with PtP40Co30 (0.15 mg cm^−2^) cathodes (in blue) under 80 °C, 100 % RH, 150 kPa_abs_, and 0.5/5 L min^−1^ H_2_/air compared before (solid) and after (dashed) the 10 000 cycles of 0.6–0.95 V 3s/3s accelerated degradation test compared to that of a reference MEA with cathode loading of 0.4 mg cm^−2^ (in black). b) Mass activity of PtP40Co30 versus reference Pt before and after AST evaluated under 80 °C, 100 % RH, 150 kPa_abs_, and 0.5/5 L min^−1^ H_2_/O_2_ at 0.9 V (iR free). c) Representative cyclic voltammetry of PtP40Co30 cathode before and after AST as compared to that of the reference Pt cathode. The roughness factor decrease is 25% for PtP40Co30 (74(BoL)/55 (EoL) cm^2^_Pt{HUPD}_/cm^2^) and 43% for the reference electrode (210(BoL)/120 (EoL) cm^2^_Pt{HUPD}_/cm^2^).

To further improve the mass activity of the strained Pt catalyst, passivation gas incorporated atomic layer deposition (PALD)^[^ ^53^ ^]^ was applied. The effect of using CoO*_x_* is also observed using the PALD route with respect to increased specific activity (Figure S9, Supporting Information). According to XRD, this route also results in higher portion of strained Pt than those from Pt20Co30 (Figure S10, Supporting Information). In addition, we optimized the membrane that further improved the mass activity at low current density (LCD) over Pt20Co30 (a full set of comparison included Table S1, Supporting Information). As a result, we achieved an average mass activity of ≈0.6 A mg_Pt_^−1^ (linearity demonstrated at various loadings as shown Figure S11, Supporting Information) for the strained catalyst deposited with 40 cycles of Pt with PALD on top of 30 cycles of CoO*_x_*, denoted as PtP40Co30 in **Figure** **4**. Nevertheless, the benefit from the membrane on the activity at the LCD does not extend to the high‐current‐density (HCD) region (Figure S12, Supporting Information). By using a carbon support with a preferable pore structure, the mass activity was pushed further close to 0.8 A mg_Pt_^−1^ as showcased by the CMK‐3 case (Figure S13, Supporting Information). However, this carbon support is not appropriate for HCD operation due to the large sizes of primary carbon particles that may have added mass transport impedance. Densely packed Pt NPs in the pore structures can possibly make water removal more difficult. The discussion below is restrained to Ketjenblack as the support. With ALD‐MEA, we have screened multiple carbon supports and ionomers and present the performance of the PtP40Co30 ALD‐MEAs with best class low loading beginning of life (BoL) performance in Figure 4a. Mass activity has arrived at a convergence within a wide range of mass loadings as showcased in Figure S14, Supporting Information. The performance of the MEA with strained catalyst is compared with that of a reference commercial Pt MEA with a higher loading whose polarization curve corresponds to the limit of what can be achieved on the HCD end with an equivalent membrane resistance. For achieving an equivalent activity in the H_2_–air MEA, a mass loading of 0.15 mg cm^−2^ is required for PtP40Co30, further reduced from that reported in Figure 2e. At an automotive relevant low loading of 0.12 mg_Pt_ cm^−2^, the ALD‐cathode demonstrated a state‐of‐the‐art MEA performance with a rated power density of 0.11 g kW^−1^ (Figure S15, Supporting Information). These ALD‐MEAs have also demonstrated good stability. After Pt dissolution accelerated stability tests (AST), only 10 mV is lost at 0.8 A cm^−2^ as shown by Figure 4b, demonstrating an impressible stability. The end‐of‐life mass activity remains at 0.39 A mg_Pt_^−1^ (Figure S11, Supporting Information), mostly due to ripening of the particles (shown by Figure S16, Supporting Information). In the major span of the polarization curve, the loss is within 15 mV and corresponds well to the 33% loss of mass activity. Catalyst design strategies such as using underlayers and selective decorations,^[^ ^59^ ^]^ that are feasible with ALD, can be further considered for preventing the structural reconstruction of Pt‐based catalyst under the real fuel cell test conditions, especially in the potential cycling. For HCD performance and stability, more attention should also be paid to factors beyond the bare catalysts, such as the support and ionomer. Figure 4c contrasts the roughness factor of the PtP40Co30 ALD cathode with that of the reference Pt, further confirming that the improvement in the MEA level is primarily attributable to an enhancement of the intrinsic activity.

### 2.5 Catalyst Challenges for High‐Power‐Density Fuel Cells

**Figure 5:**
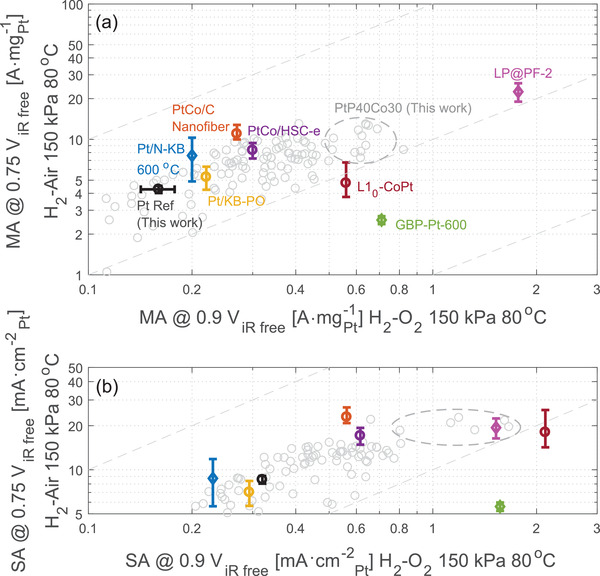
Mass activity of Pt catalysts at low‐current‐density (0.9 V iR‐free in H_2_–O_2_) and high‐current‐density (0.75 V iR‐free in H_2_–air) regions. ALD‐cathodes of various configurations are plotted as gray circular dots with the dataset for PtP40Co30 circled by the gray dashed ellipse. All data points correspond to activity at 80 °C, under a differential condition with high stoichiometry. For data points not measured at that 150 kPa_abs_ air, corrections are made for H_2_–air data by considering an apparent kinetic order from 0.5 to 1 and for H_2_–O_2_ data by extending the reported Tafel plot to 0.8–0.78 V iR‐free. Estimates made according to H_2_–O_2_ data are plotted in diamonds for the grain boundary porous platinum (GBP‐Pt @0.2 mg cm^−2^),^[^ ^67^ ^]^ Pt‐alloy/Pt‐free dual‐catalysts (LP@PF‐2@0.035 mg cm^−2^),^[^ ^31^ ^]^ and Pt on nitrogen‐modified Ketjenblack (Pt/N‐KB @0.11 mg cm^−2^).^[^ ^43^ ^]^ Reported H_2_–air data and estimates according to reported H_2_–air data are plotted in circles for PtCo/C nanofiber (@0.1 mg cm^−2^),^[^ ^70^ ^]^ PtCo on HSC‐e support (@0.06 mg cm^−2^),^[^ ^69^ ^]^ Pt/Ketjenblack by polyol reduction (Pt/KB‐PO) (@0.06 mg cm^−2^),^[^ ^68^ ^]^ and hard magnet core–shell L1_0_‐CoPt/Pt (@0.1 mg cm^−2^).^[^ ^6^ ^]^

There have been tremendous efforts in the scientific community to enhance the mass activity of ORR catalysts, as recently reviewed by Huang et al.^[^ ^60^ ^]^ in an exhaustive report. Most catalyst research focused on the RDE performance, and few have demonstrated the catalysts’ activities in the MEA, with even fewer proving fuel cell performances with practical relevance. A good portion of MEA‐level works has been directed toward exerting activity and stability of powder catalysts. A remarkable benchmark has been established by Shao‐Horn et al. in 2015 on Pt–Ni catalysts^[^ ^61^ ^]^ and an industrial perspective has been given by Ye et al. in 2017.^[^ ^62^ ^]^ Since the recommendation by Kongkanand et al. in 2016^[^ ^38^ ^]^ to use a differential flow condition to eliminate variations among flow fields, more works have been reporting MEA relevant catalyst performance in similar conditions. Despite often reported at different oxygen partial pressures, there are more data points on performance evaluated at the same temperature, 80 °C, which enables the discussion below. For practical energy applications of fuel cells, power density is one of the ultimate pursuits. Mass activity reported at 0.9 V iR‐free in an H_2_–O_2_ MEA corresponds to tens of mA cm^−2^ as opposed to a few A cm^−2^ when the power performance is evaluated. This corresponds to a notable difference of two orders of magnitude. For the efficiency of energy conversion with fuel cells, power is rated around or above 0.65 V. Considering iR losses, we set 0.75 V iR‐free as a benchmark point for reporting HCD performance in **Figure** **5**. The choice of this voltage also considers that the current density for low‐loaded electrodes (≈0.1 mg cm^−2^) does not introduce significant transport losses that render iR correction insufficient. For performance from the literature that is not evaluated at H_2_–air, we adopted their H_2_–O_2_ performance and estimated their equivalent performance as 0.78–0.8 V iR‐free considering an apparent reaction order from 0.5 to 1. Variations in back‐pressure are corrected with the same reaction order range. The reference Pt electrode has its activity well‐benchmarked with values reported in the literature^[^ ^61^, ^63^, ^64^, ^65^, ^66^ ^]^ over a loading range of <0.1 to 0.4 mg cm^−2^. Performances of the studied ALD‐electrodes with different builds are showcased in the background to establish a baseline goal of enhancing HCD performance by using active catalytic systems that excel at LCD. It is noted that not all the data points follow the trend, some electrodes show high LCD but low HCD performance. An example from the literature is the coordination site optimized Pt particles by Cheng et al.,^[^ ^67^ ^]^ which fall into the same dilemma of not capable of delivering HCD performance despite a remarkable LCD activity reported. Overall, our optimization efforts have landed on the PPt40Co30 samples with more than tripled LCD and doubled HCD performance compared to the reference Pt electrodes. A mass activity around 10 A mg_Pt_^−1^ is achieved at 0.75 V iR‐free under H_2_–air 150 kPa_abs_. Several state‐of‐the‐art catalytic systems have approached similar HCD performance (including credible but not printed results as compiled in Figure S18, Supporting Information). The strategies of Pt‐ionomer relative distribution tuning with carbon support modification^[^ ^43^, ^68^, ^69^ ^]^ and agglomerate engineering^[^ ^70^ ^]^ have shown promises in enhancing both LCD and HCD performance. Catalytic activity is less relevant to HCD than LCD as the activation energy barrier is significantly reduced by the overpotential applied at a lower voltage.^[^ ^71^ ^]^ Catalyst and oxygen utilization are also important factors that can limit the transfer of activity from LCD to HCD.^[^ ^72^ ^]^ Despite a wide range of reported LCD activities, HCD performance converges to around 10 A mg_Pt_^−1^. For an electrode with 0.1 mg cm^−2^ loading, this can correspond to 1 A cm^−2^ above 0.7 V (assuming the areal resistance is between 40 and ≈50 mOhm cm^2^). The dual‐catalysts electrodes developed by Chong et al.^[^ ^31^ ^]^ demonstrate an exception, with normalized mass activity benefited by contributions from both platinum‐based and platinum‐free catalysts. Their electrodes with ultralow platinum loading exhibit compromised power performance; however, the effectiveness of shrinking the denominator has proven the promises in enhancing platinum utilization. Among the many strategies in this line, one can enhance ECA by further increasing the ratio of surface platinum atoms that are catalytically active, for example, by optimizing catalyst geometry, especially given that ALD can be utilized to synthesize atomic scale catalyst down to single‐atom active sites.^[^ ^73^, ^74^ ^]^ With innovations on ALD chemicals, especially the Pt ALD precursors,^[^ ^75^, ^76^ ^]^ and deeper understanding and innovation of ALD deposition processes,^[^ ^77^, ^78^ ^]^ ORR activity normalized by mass or cost are expected to be improved further.

## 3 Conclusion

We have demonstrated a strained Pt catalyst with an enhanced catalytic activity for ORR in both RDE and MEA. The catalyst was fabricated by sequential ALD deposition of cobalt oxide and Pt on carbon supports, followed by acid leaching that removed the cobalt oxide template almost entirely. This process can be scaled via either spatial ALD reactors^[^ ^79^ ^]^ or powder ALD catalysts manufacturing in the fluidized bed,^[^ ^80^, ^81^, ^82^ ^]^ with others compatible to conventional catalysts application techniques. The material synthesis process resulted in a strain on Pt particles, which was optimized by systematically varying the dimensions of Pt and CoO*_x_* via ALD cycle numbers. Roughly twice the specific activity improvement over that of Pt reference was transferrable to the MEA level. A compressive strain in the Pt—Pt lattice was observed by both EXAFS and HRTEM in with a negligible Pt—Co interaction. Considering the negative correlation between specific activity and the Co content, the performance enhancement can be mostly attributed to the Pt lattice strain. With PALD, we pushed the mass activity to 0.6 A mg_Pt_^−1^ on a Ketjenblack carbon support and even close to 0.8 A mg_Pt_^−1^ on CMK‐3. The Ketjenblack‐supported catalyst additionally demonstrated impressive durability. The translation from active catalysts to high‐performance fuel cell is not always guaranteed and requires engineering on several aspects of the electrodes. More attention shall be paid on the behavior and reaction mechanism of catalytic systems at HCDs to enable low‐Pt‐loaded fuel cells with high power density.

## Conflict of Interest

The authors declare no conflict of interest.

## Author Contributions

S.X. and Z.W. contributed equally to this work. S.X. conceived the catalyst design and explored the synthesis methods with Z.W. and Q.T. in the RDE. S.X., Z.W., and S.D. optimized MEA performance with the guidance of D.H. and G.H.; S.X., D.U.L., and M.O. optimized MEA test setup and protocol with the guidance of D.H., G.H., and S.K.; S.X., Y.L., A.L.D., P.S., J.S.L.P., and P.V. performed the material characterization and analysis. O.V., V.V., T.D.S., and J.E.M. performed theoretical investigation of the structure of catalytic activity. S.X., Z.W., S.D., P.S., J.T., and F.B.P wrote the manuscript.

## Supporting information

Supporting Information

## Data Availability

The data that support the findings of this study are available from the corresponding author upon reasonable request.
