# Supplementary material for: Direct Integration of Strained‐Pt Catalysts into Proton‐Exchange‐Membrane Fuel Cells with Atomic Layer Deposition
Source: Adv Mater. 2021 Jun 10;33(30):2007885. doi: 10.1002/adma.202007885 (PMC11468935; doi:10.1002/adma.202007885)
Supplement: Supplementary file 1 — Supporting Information [file ADMA-33-2007885-s001.pdf]

© 2021 Wiley-VCH GmbH

ADVANCED MATERIALS

Supporting Information

for *Adv. Mater.,* DOI: 10.1002/adma.202007885

Direct Integration of Strained-Pt Catalysts into Proton-Exchange-Membrane Fuel Cells with Atomic Layer Deposition

Shicheng Xu, Zhaoxuan Wang, Sam Dull, Yunzhi Liu, Dong Un Lee, Juan S. Lezama Pacheco, Marat Orazov, Per Erik Vullum, Anup Lal Dadlani, Olga Vinogradova, Peter Schindler, Qizhan Tam, Thomas D. Schladt, Jonathan E. Mueller, Sebastian Kirsch, Gerold Huebner, Drew Higgins, Jan Torgersen, Venkatasubramanian Viswanathan, Thomas Francisco Jaramillo, and Fritz B. Prinz**

© Copyright 2020. WILEY-VCH GmbH.

Supporting Information

Direct Integration of Strained-Pt Catalysts into Proton-Exchange-Membrane Fuel Cells with Atomic Layer Deposition

Shicheng Xu‡, Zhaoxuan Wang‡, Sam Dull, Yunzhi Liu, Dong Un Lee, Juan S Lezama Pacheco, Marat Orazov, Per Erik Vullum, Anup Lal Dadlani, Olga Vinogradova, Peter Schindler, Qizhan Tam, Thomas D. Schladt, Jonathan E. Mueller, Sebastian Kirsch, Gerold Huebner, Drew Higgins, Jan Torgersen, Venkatasubramanian Viswanathan, Thomas Francisco Jaramillo, Fritz B. Prinz**

Section S1. Experimental Details

Sample preparation

Trimethyl(methylcyclopentadienyl) platinum (IV) ($MeCpPtMe_3$, Strem Chemicals) and Bis(cyclopentadienyl) cobalt(II) (Sigma-Aldrich) were used as Pt and Co precursors for ALD. Ozone was used as oxidant. Catalyst for RDE was deposited onto GC disks with one side polished (diameter 5mm, thickness 4mm, SIGRADUR®, HTW chemicals, RMS roughness = ~1.9 nm). For MEA cathode preparation, Ketjen Black was deposited onto a Sigracet 29BC gas diffusion layer (GDL) by filtrating carbon dispersion through the GDL, which resulted in a 2-4 μm carbon layer (~0.1–0.2 mg/cm^2). Co precursor cylinder was heated to 65 ^oC and cobalt oxide deposition was conducted at a reactor temperature of 160 ^oC. For normal ALD of Pt, precursor cylinder was heated to 78 ^oC and reactor temperature was 160 ^oC. For Pt PALD, the reactor temperature was 120^oC. For GC samples, Pt loadings were determined by inductively coupled plasma mass spectrometry; and for GDE samples, the mass was quantified with X-ray fluorescence (SPECTRO XEPOS spectrometer HE). The membrane electrode assembly (MEA) was constructed by the compressing the GDE against an anode coated membrane (0.1 mg/cm^2 Pt) at room temperature at 0.4 MPa. The samples used the same type of membrane and anode unless specified.

Electrochemical testing

ORR catalyst was tested in a three-electrode cell equipped with RDE as working electrode, pure platinum wire as counter electrode and reversible-hydrogen electrode (RHE) as reference electrode. Electrolyte was 0.1 M $HClO_4$ solution prepared by diluting 70% $HClO_4$ aqueous solution (Merck, Suprapur) with ultrapure deionized water (18.2 MΩ·cm, TOC < 5 ppb). The electrochemical testing related to ORR was conducted by Gamry potentiostat (Interface 1010 E) and all gases for testing were ultrapure grade, including 99.9999% Ar, 99.999% O_2, and 99.999% H_2(Praxair). RDE testing protocol is according to accepted method that previously published.[1] Prior to electrochemistry measurement, 30 min acid leaching procedure is studies, using pH=3, pH=4 $HClO_4$ solution(prepared by diluting 70% $HClO_4$ aqueous solution with ultrapure deionized water). The results comparison for pH=3, pH=4 acid leaching along with no pre-leaching is shown in Figure S2, and pH=4 is chosen as the solution for acid leaching with a most pleasant performance. The activation process is studied under 100 cycles of cyclic voltammetry (CV) mode with sweeping rate of 500 mV/sec, voltage range of 0.025 to 0.8, 1, 1.2 V *vs.* RHE respectively, electrode rotation of 2500 rpm, and in oxygen-free electrolyte sufficiently purged by Ar gas. Based on the results (Figure S19), no activation procedures have been applied to the Pt/CoOx samples. ORR activities were measured by background linear sweeping voltammetry (LSV) mode with sweeping rate of 20 mV/sec, voltage range of -0.01 to 1.0 V *vs.* RHE, and electrode rotation rate of 1600 rpm, with electrolyte purged by oxygen for more than 10 mins. Kinetic current density (J_k) was obtained from ORR corrected by background LSV (voltage range of -0.01 to 1.0 V *vs.* RHE, sweeping rate of 20 mV/sec, electrode rotation rate of 1600 rpm in Ar-saturated electrolyte) and electrolyte resistance (by electrochemical impedance spectroscopy (EIS)). Specific activity (SA, J_k normalized by the electrochemical active surface area (ECSA)), and the mass activity (MA, J_k normalized by Pt loading) were calculated. ECSA was measured after residual oxygen in the electrolyte is subsequently eliminated with Ar, using 3 cycles of CV with sweeping rate of 50 mV/sec, voltage range of 0.025 to 1 V *vs.* RHE, electrode rotation of 0 rpm.

MEA testing was done in a Scribner 840 fuel cell testing system using a Greenlight 50 cm^2 research cell fixture. For activity measurements, O_2 (99.993%, Praxair) and H_2 (99.999%, Praxair) were fed at the flow rate and back pressure as specified in the data presented. For each cell potential (0.82 V, 0.85 V, 0.88 V, 0.89 V, and 0.90 V), current densities were measured at the end of 5 min.[2-4] Polarization curves were taken in clean dry air with currents held for 3 min, per the US DOE protocol.[5] The cell resistance was taken from the high-frequency x-intercept of an electrochemical impedance Nyquist plot measured at 0.25 A in oxygen.

Material Characterization

The morphology and crystal structure of the samples were examined by high-resolution transmission electron microscopy (HRTEM) image analysis using an aberration-corrected transmission electron microscope (FEI Titan ETEM 80-300) at 300 kV acceleration voltage. The high-resolution images were calibrated using a Si reference sample viewing from [110] zone axis. All HRTEM images were taken under the dose rate less than 5000 $e-/Å^2s$ within 10 secs, to prevent possible alternation of nanoparticles induced by electron beam.

X-ray absorption spectroscopy experiments were performed at the Pt L3 edge at beamline 7-3 at the Stanford synchrotron Radiation Laboratory. A double crystal monochromator was used to select the energy, with the second crystal was detuned 30% to remove second order harmonics. A Pt metal foil was used for calibration and set to a value of 11564 eV. Data processing was performed using the Horae analysis suite.[6]

Section S2. Supplementary data and results

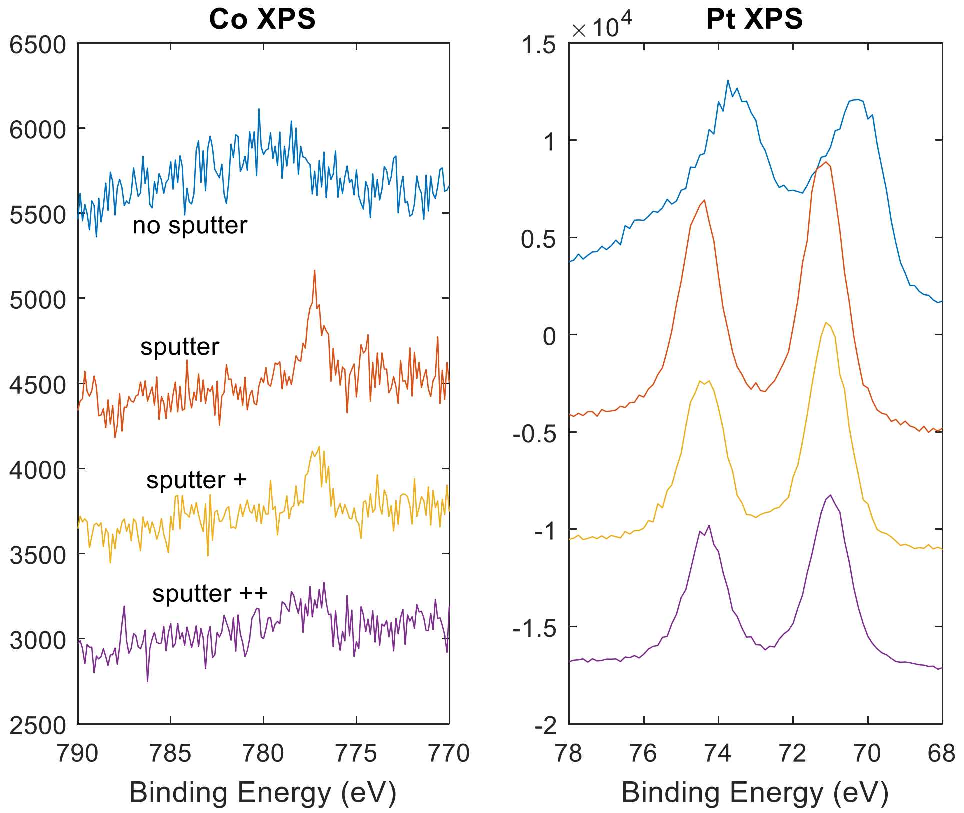

Figure S1. XPS depth profile of Pt/CoOx deposited (prior to acid treatment) on a silicon substrate. There is significant amount of Pt on the sample surface with detectable cobalt whose signal gets more significant after argon sputtering. This indicates that Co is beneath the Pt top layers.

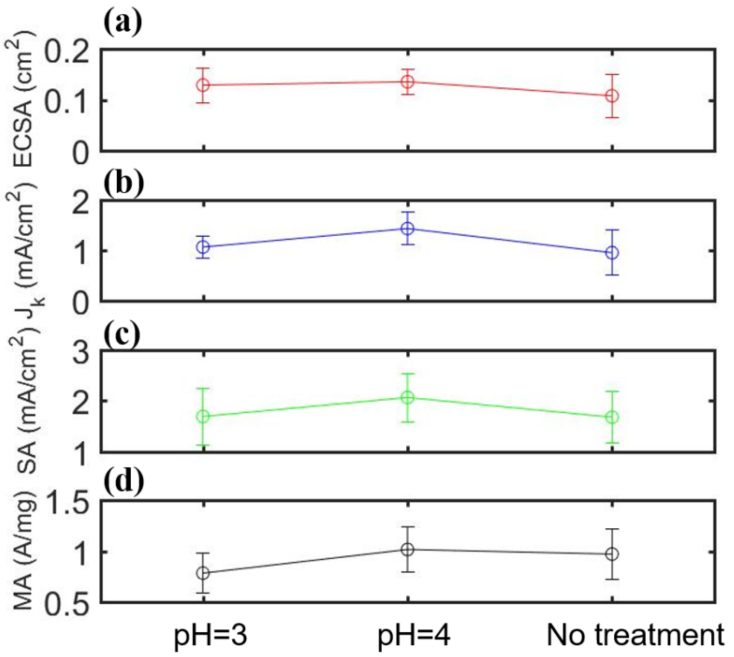

Figure S2: Effects of pre-leaching of PtCo catalyst in various concentration of $HClO_4$ solution on (a) ECSAs (b) kinetic current densities at 0.9 V *vs.* RHE (c) specific activities at

0.9 V *vs.* RHE (d) Pt-based mass activities at 0.9 V *vs.* RHE. Statistics based on 5 parallel samples of same ALD batch. Note that no treatment leads to loss of Pt despite retained mass activity.

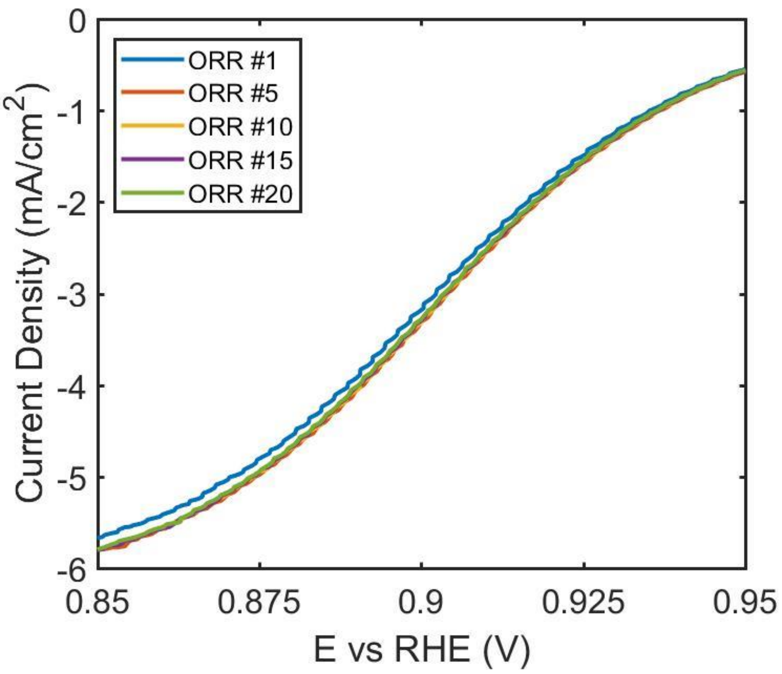

Figure S3: Convergence of linear sweep voltammetry for evaluating the ORR activity of a typical catalyst. Data at #20 scan is taken for evaluation while convergence usually arrives at scan #10.

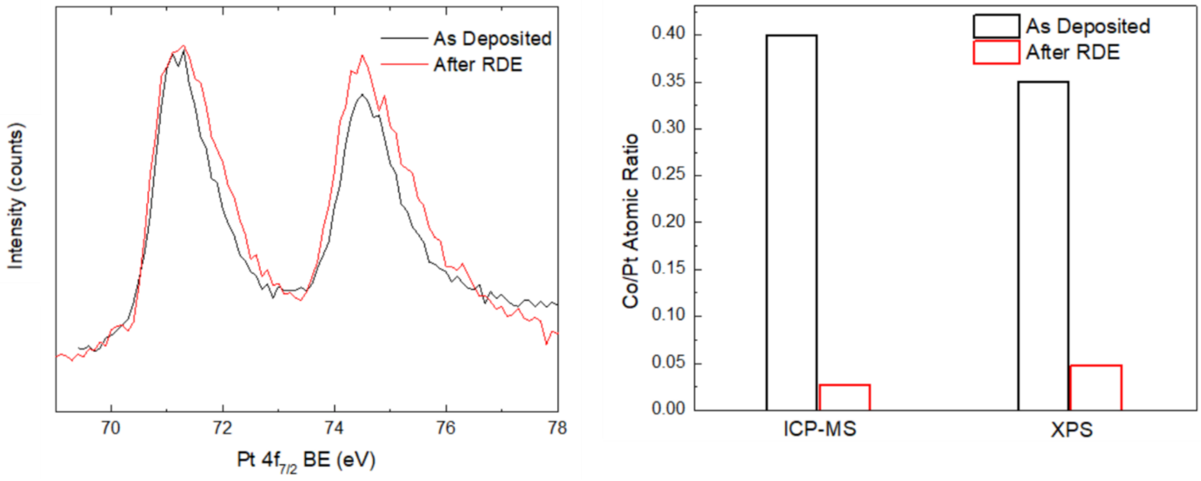

Figure S4. Pt4f$_{7/2}$ XPS spectra and cobalt to platinum atomic ratio confirmed by XPS and ICP-MS before and after RDE testing. Most cobalt component leached out and only a minute amount cobalt left in the catalysts.

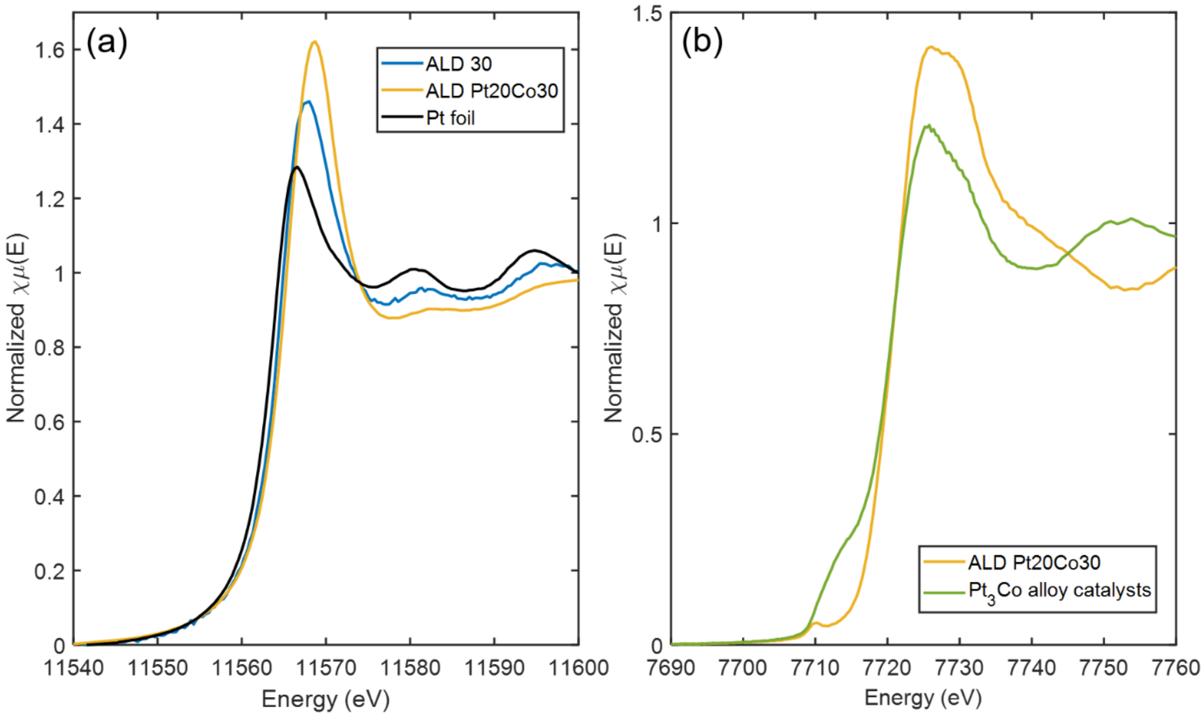

Figure S5. XANES spectra of a) Pt30, Pt20Co30, Pt foil at Pt L_3 edge, and b) Pt20Co30 and a commercial Pt_3Co alloy catalyst at Co K edge.

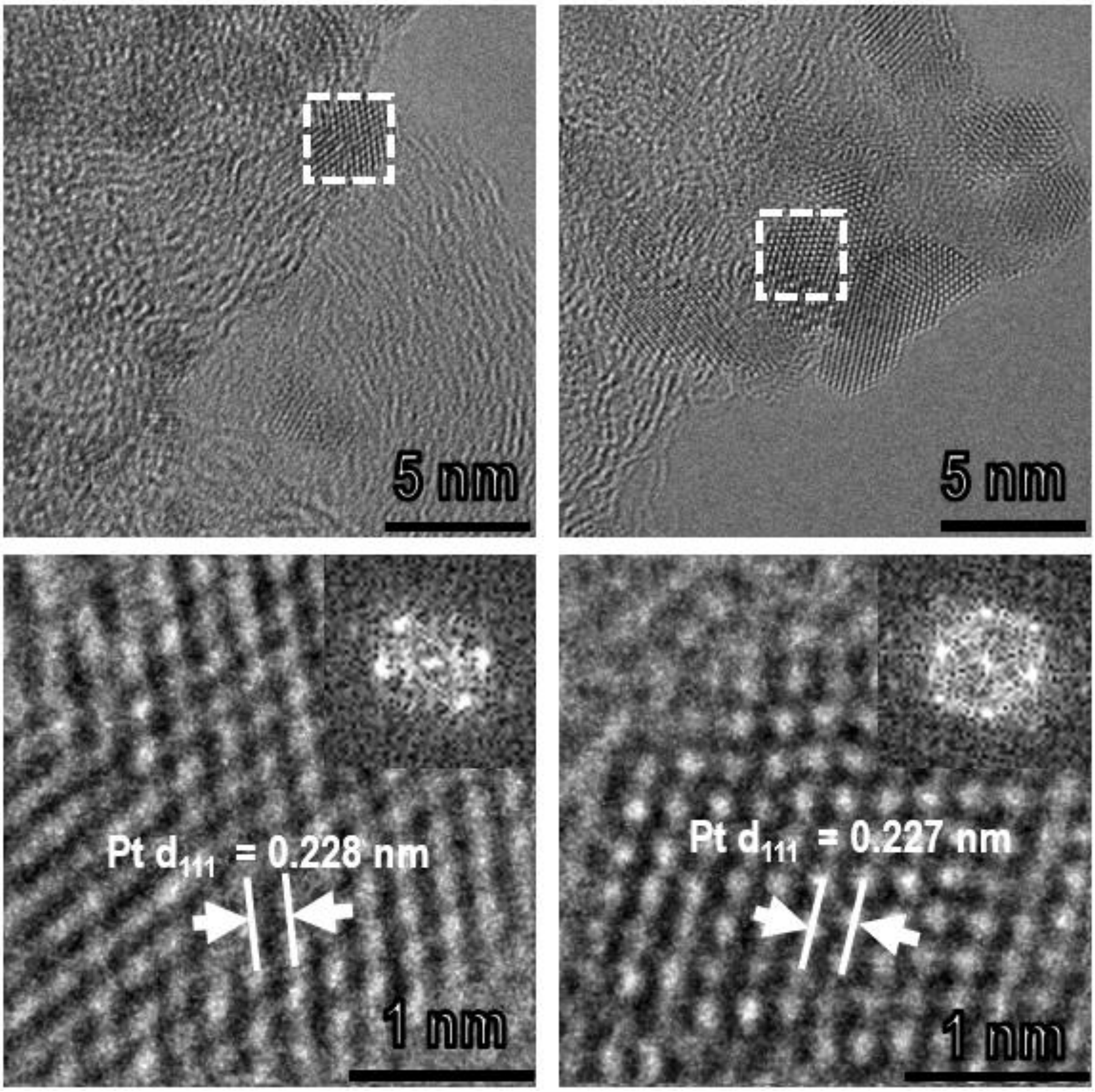

Figure S6. HRTEM images of the Pt/C samples (TEC10E50E, 46.6 wt%) with (111) plane spacing measured on particles (on the left and right columns respectively) viewed from a zone axis of [110].

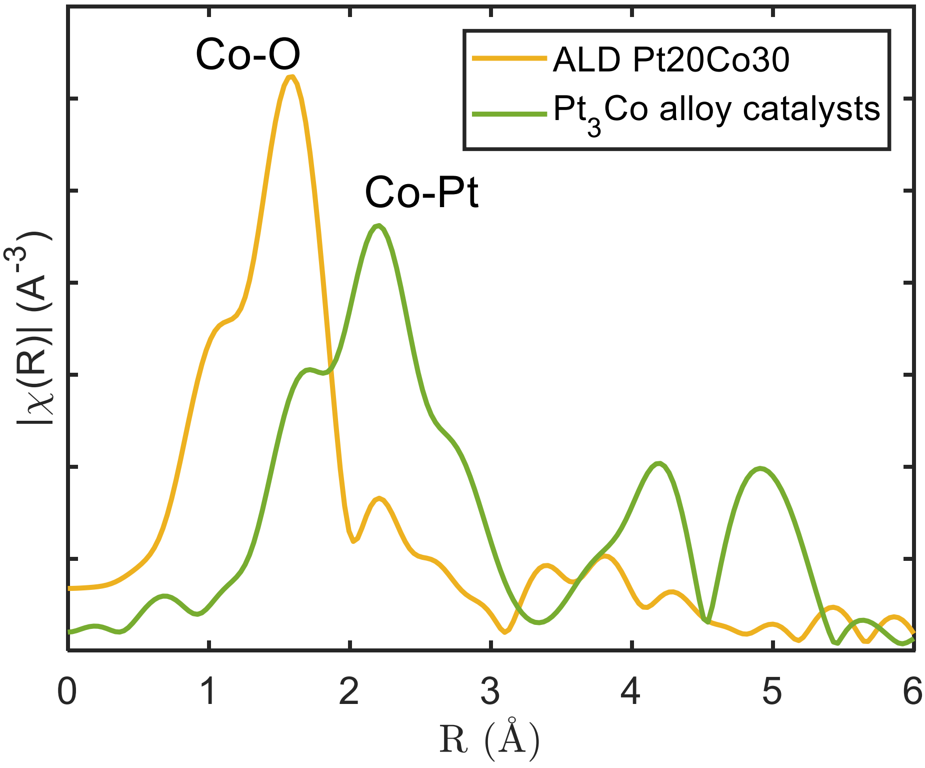

Figure S7. Co K edge EXAFS spectra of Pt20Co30 after the BOL MEA tests compared to that of a commercial Pt_3Co alloy catalyst.

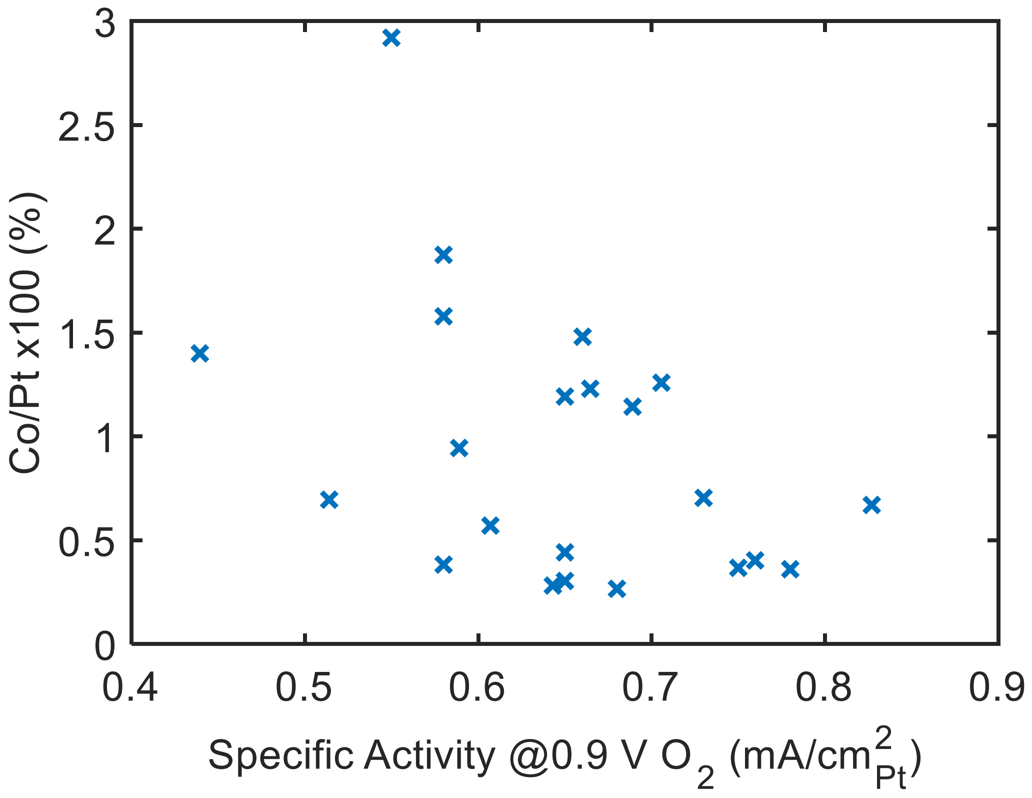

Figure S8. Specific activity of the strained Pt catalysts in the MEA vs their cobalt content in atomic ratio.

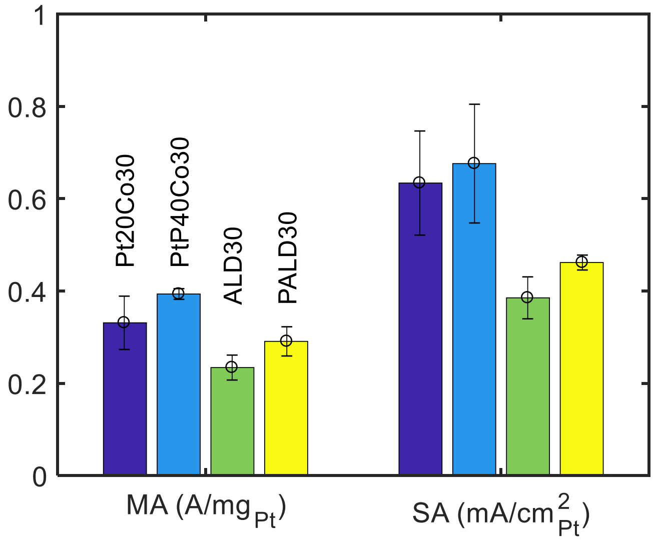

Figure S9. Mass activity and specific activity @ 0.9 V iR free of ALD30, PALD30, Pt20Co30, and PtP40Co30 in the MEA with the membrane same as that used in Figure 2. The average values and error bars were obtained as the standard deviation of measurements from three to five replicate samples.

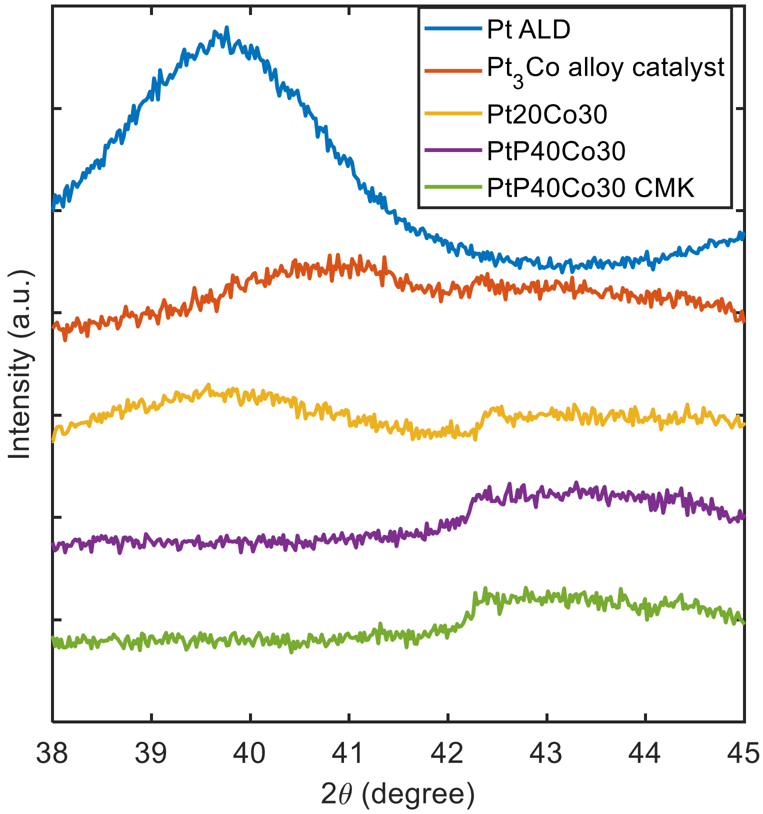

Figure S10. XRD of ALD Pt, Pt20Co30, PtP40Co30 on KB and CMK, and a commercial Pt_3Co alloy catalyst. The shift of Pt(111) to higher degrees reflect a compressive strain in the samples. Peaks shifted from 39.7^{o} to around 42.5 ^{o} can correspond to a d-spacing change of 5%.

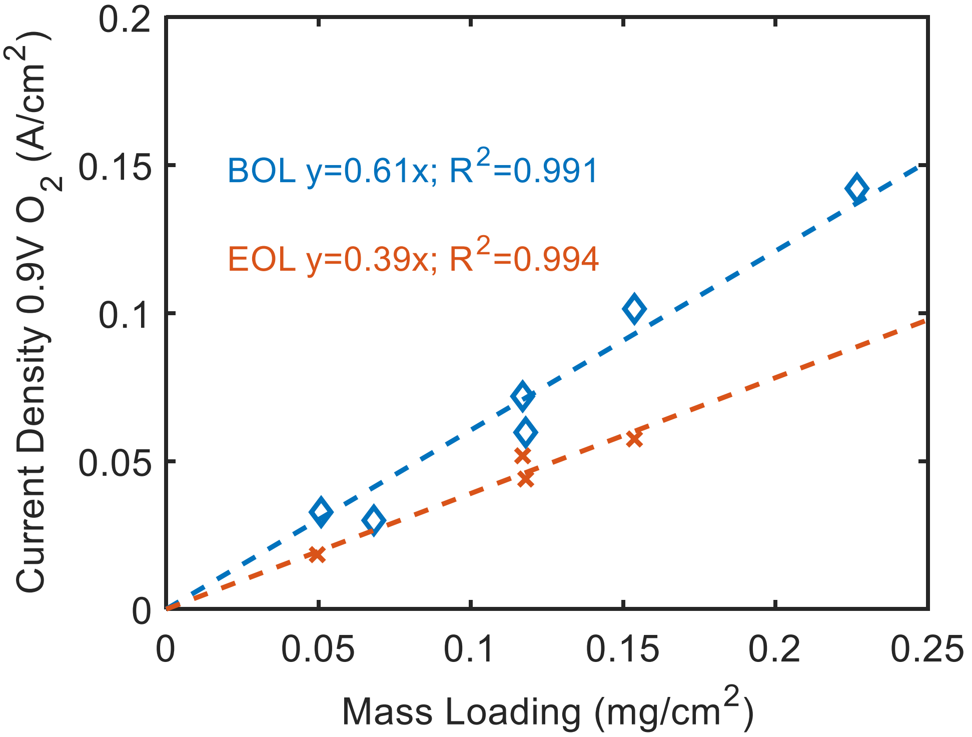

Figure S11. Mass activity quantification using PtP40Co30 cathodes at various loading. Data of both BOL and EOL as well as their linearity are presented.

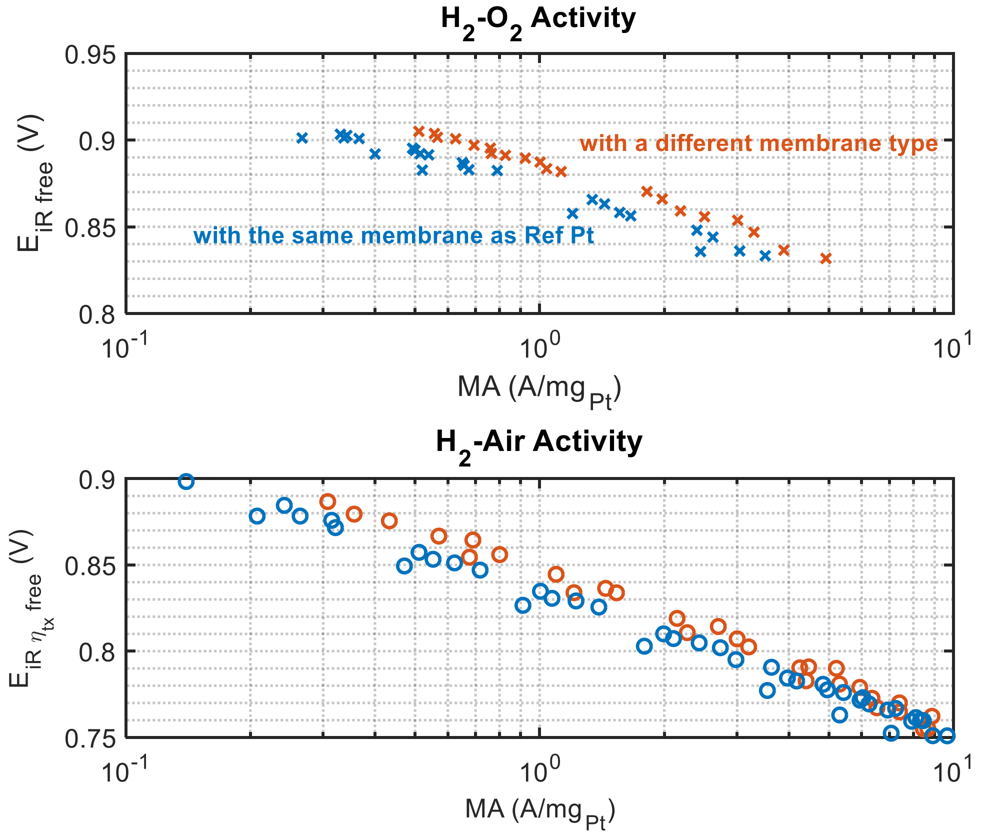

Figure S12. Mass activity under H_2-O_2 and H_2-Air conditions of PtP40Co30 cathodes at various loading (0.05 ~0.23 mg/cm^2) as a result of change in the type of membrane. The results converge at the low voltage end.

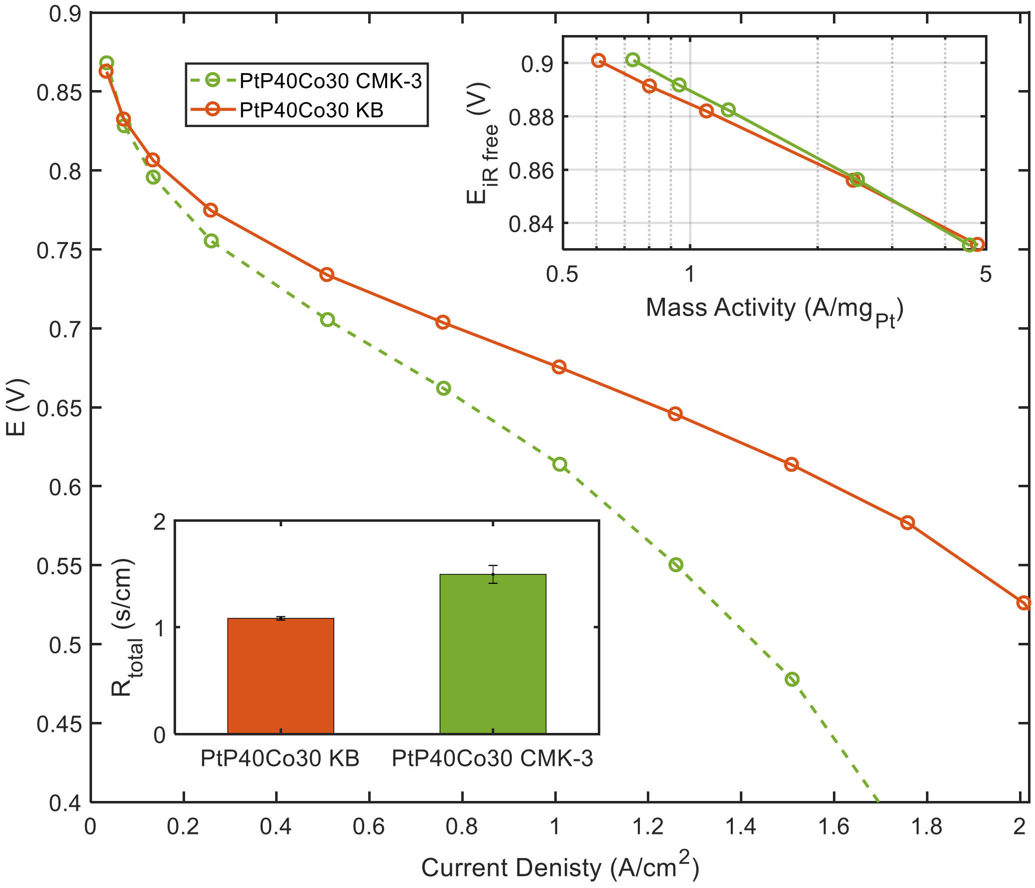

Figure S13. H_2-Air pol curve of PtP40Co30 cathodes using CMK-3 and KB as carbon support. Pt loadings are 0.05 mg/cm^2 for both samples. The mass activity is presented by the Tafel plot as an inset on the top right corner.

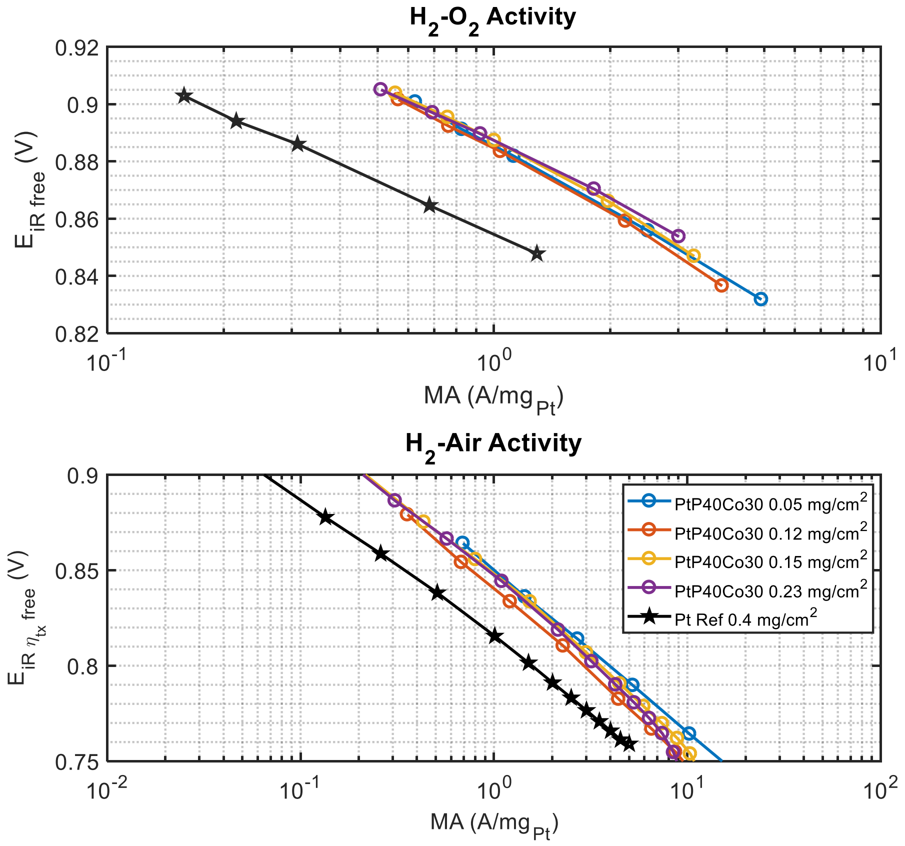

Figure S14. Mass activity under H_2-O_2 (top) and H_2-Air (bottom) conditions of PtP40Co30 cathodes at various loading compared to that of the reference electrode. The test conditions are same as the one applied in Figure 4.

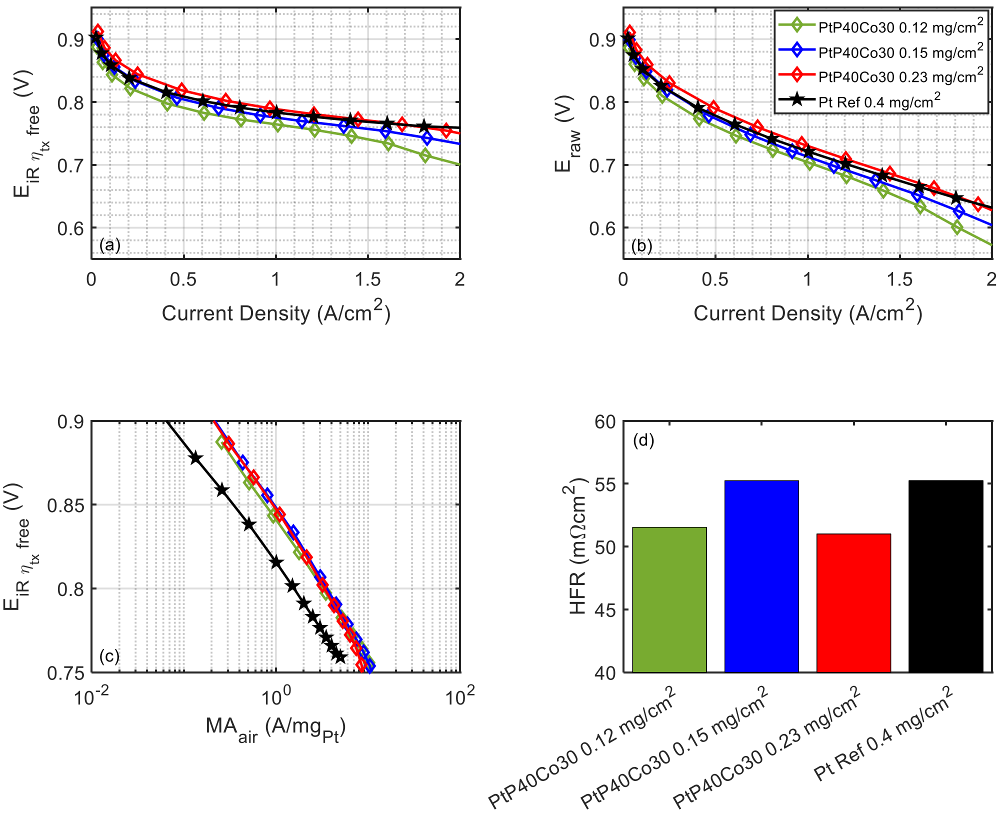

Figure S15. In addition to the PtP40Co30 (0.12 mg/cm^2) and the Reference Pt (0.4 mg/cm^2) in **Figure 4**, PtP40Co30 electrode with higher mass loading are compared in a) iR and transport corrected polarization curve, b) non corrected polarization curve, c) mass activity in the H_2-Air measurement with the same test conditions as **Figure 4**, along with (d) the high frequency resistance of these four MEAs. Equivalent LCD and HCD performance to the reference Pt electrode have arrived when PtP40Co30 mass loading reaching 0.15 mg/cm^2 and 0.23 mg/cm^2, respectively.

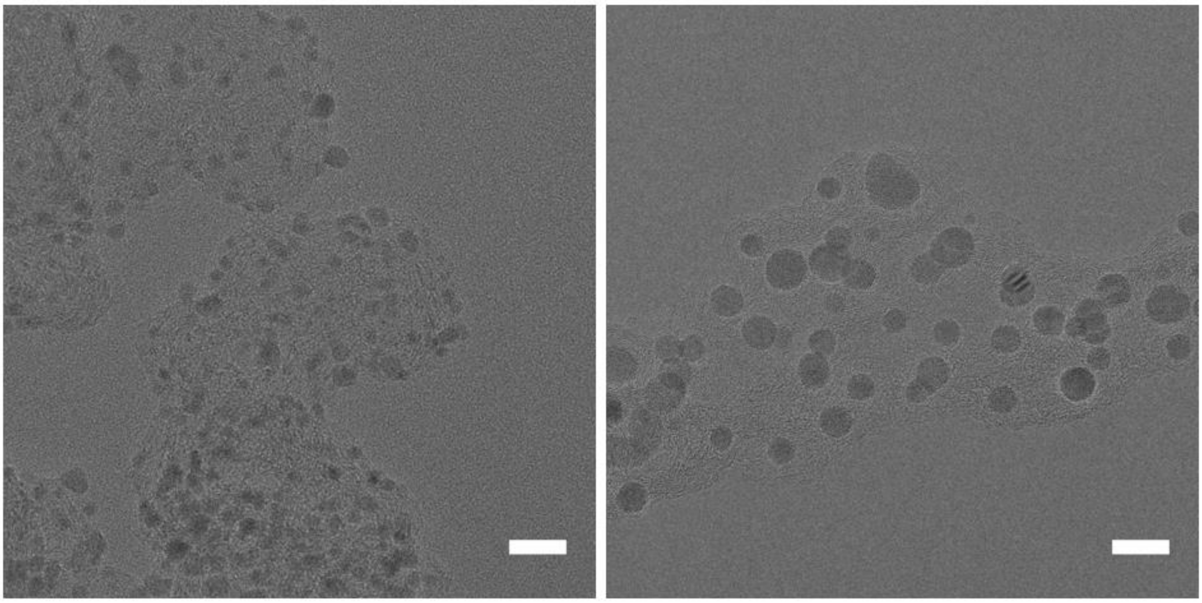

Figure S16. PtP40Co30 nanoparticles before (left) and after (right) AST indicate particle ripening. The scale bar represents 10 nm.

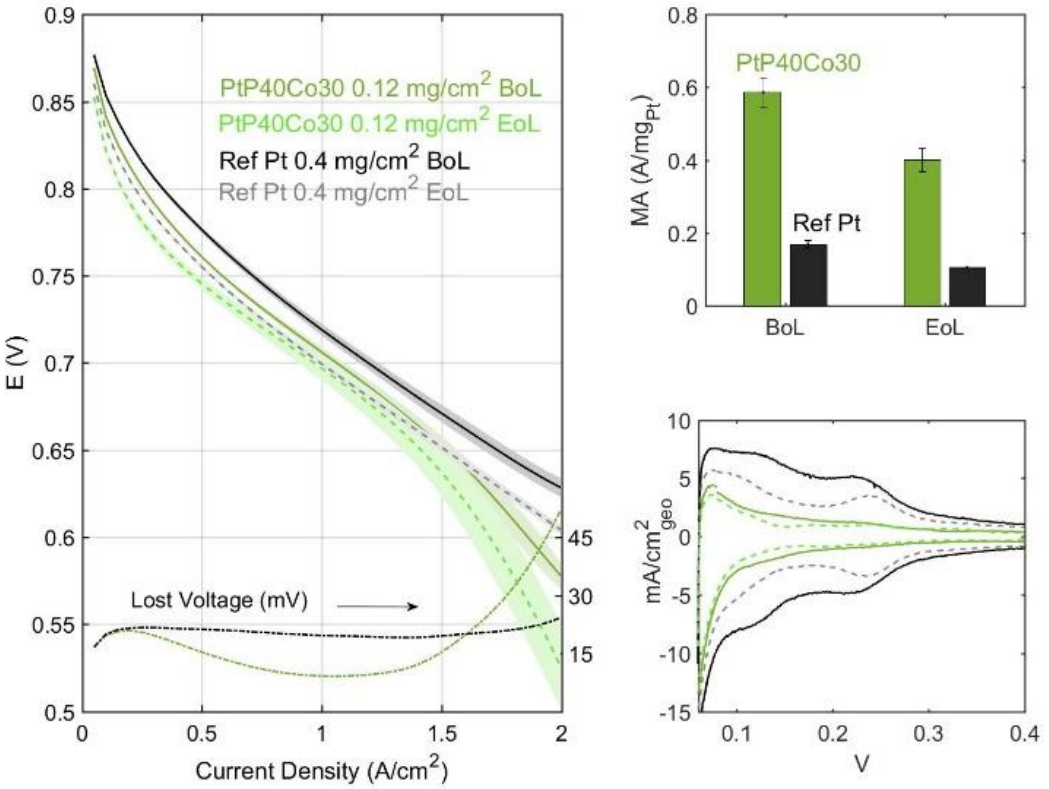

Figure S17. MEA performance of low-loaded strained Pt catalyst. (a) Fuel cell performance of 5 cm^2 MEAs with PtP40Co30 (0.12 mg/cm^2) cathodes (in green colors) under 80 ^oC, 100 % RH, 150 kPa_{abs}, and 0.5/5 L/min H_2/air compared before (solid) and after (dashed) the 10,000 cycles of 0.6-0.95 V 3s/3s accelerated degradation test compared to that of a reference MEA with cathode loading of 0.4 mg/cm^2 (in black colors). The errors are presented as shaded areas. (b) Mass activity of PtP40Co30 versus reference Pt before and after AST evaluated under 80 ^oC, 100 % RH, 150 kPa_{abs}, and 0.5/5 L/min H_2/O_2 at 0.9 V (iR free). The average values and error bars above were obtained as the standard deviation of measurement of two replicate samples for polarization curves and AST tests. (c) Representative cyclic voltammetry of PtP40Co30 cathode before and after AST as compared to that of the reference Pt cathode.

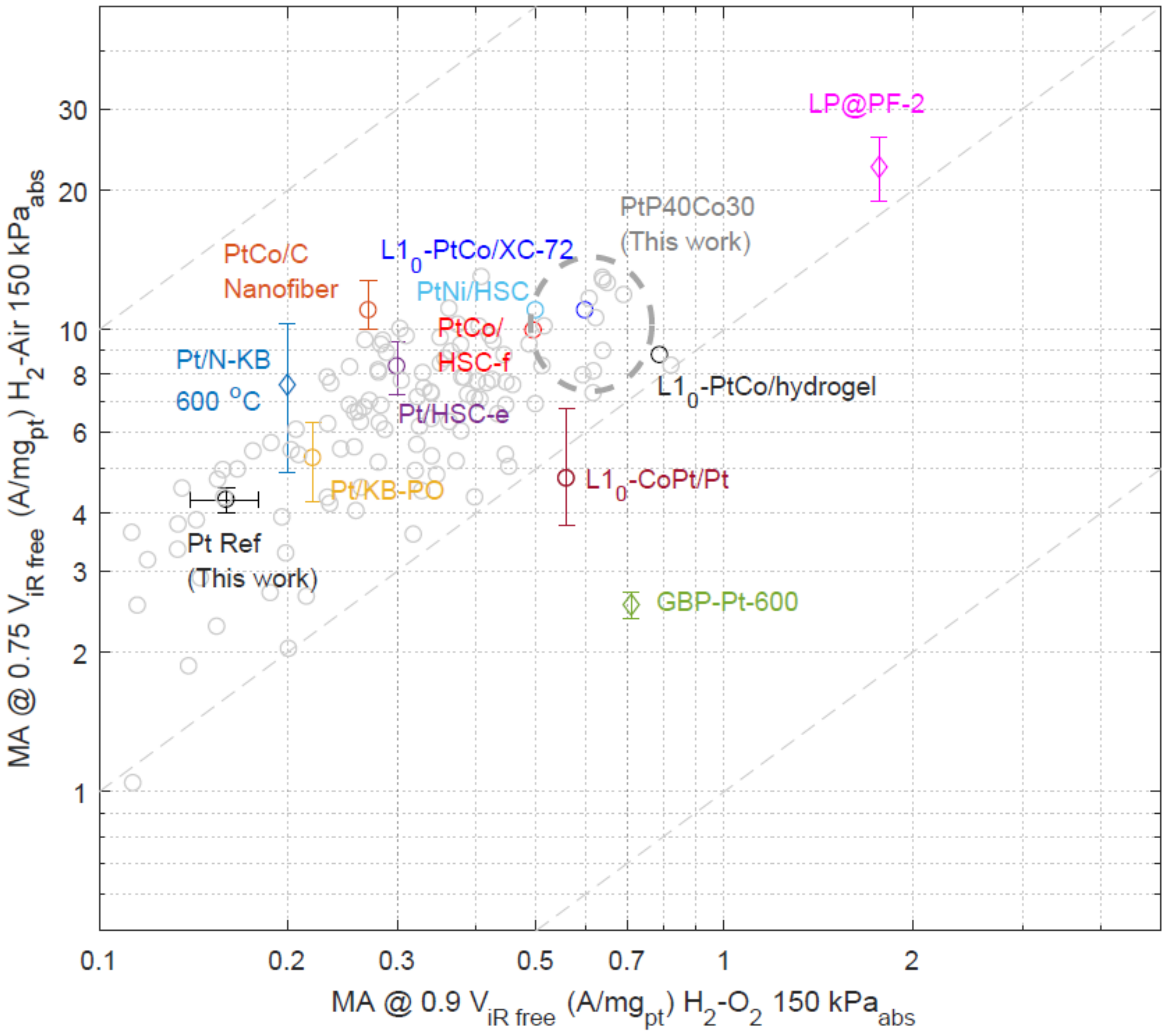

Figure S18. Mass activity of Pt catalysts at low current density (0.9 V iR-free in H_2-O_2) and high current density (0.75 V iR-free in H_2-Air) regions. In addition to Figure 5 in the main text, data points from the US DOE-Annual Merit Meetings are added: PtNi/HSC (@0.05 mg/cm^2) by Stamenkovic and Markovic, FC140, 2018 , PtCo/HSC-f (@0.1 mg/cm^2) by Kumaraguru, FC156, 2019, L1_0-PtCo/XC72 (@0.11mg/cm^2), and L1_0-PtCo/hydrogel (@0.11mg/cm^2) Spendelow, FC161, 2019.

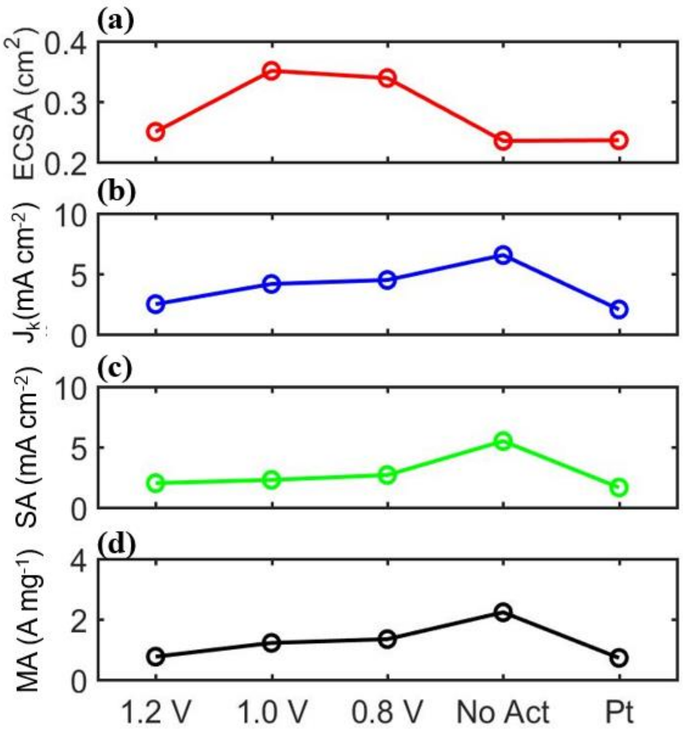

Figure S19. Effects of activation process in RDE of PtCo catalyst with various voltage range on (a) ECSAs (b) kinetic current densities at 0.9 V *vs.* RHE (c) specific activities at 0.9 V *vs.* RHE (d) Pt-based mass activities at 0.9 V *vs*. RHE.

Table S1. Mass activity (@0.9V), specific activity(@0.9V), and electrochemically active surface area determined by HUPD of catalysts discussed in this work. The cathodes evaluated with a different membrane than that of the reference electrode are denoted by -M. The average data and error bars were obtained as the standard deviation of measurement of 4-5 samples with exceptions specified in footnotes.

	MA (A mg_{Pt}^{-1})	SA (mA $cm^{-2}_{Pt\{HUPD\}}$)	ECA ($m^2_{Pt\{HUPD\}}$ g_{Pt}^{-1})	Loading (ug_{Pt} cm^{-2})
		RDE		
Pt15	0.90±0.05	1.49±0.02	62.4±1.6	1.90±0.28
Pt30Co40	2.05±0.38	3.04±0.25	68.1±15	2.03±0.32
Pt25Co40	2.10±0.90	4.31±1.02	48.2±23	1.50±0.35
Pt30Co30	1.79±0.20	2.49±0.22	43.6±8.1	2.49±0.22
Pt25Co30	1.62±0.14	3.76±0.51	44.0±8.0	2.43±0.35
Pt20Co40	1.61±0.30	2.76±0.49	62.0±23	1.43±0.21
Pt20Co30	1.07±0.20	3.34±0.55	32.0±6.4	2.06±0.12
		MEA		
Ref-Pt	0.17±0.01	0.32±0.02	53.6±1.4	
Pt30	0.23±0.03	0.38±0.04	60.9±4.1	
Pt30Co40[a)]	0.24	0.65	36.9	
Pt20Co30	0.33±0.06	0.63±0.11	52.4±4.5	
PtP40Co30	0.37±0.04	0.66±0.09	57.4±8.8	
Pt20Co30-M[b)]	0.49±0.09	1.0±0.7[b)]	61.5±29[b)]	
PtP40Co30-M[b)]	0.57±0.08	1.2±0.4[b)]	53.7±25[b)]	

a) this sample is a single measurement.
b)this type of membrane led to random crossovers and introduced errors in cyclic voltammetry measurements.

Table S2. EXAFS fitting results assuming mixture of two crystalline species: Pt and PtO. Data was fit in R space using Artemis between of 2.05 < R < 3.15 Å and Δk = 3 −11.5.

	R-factor	Pt (%)	Pt-Pt distance in Pt (Å)	Pt-Pt distance in PtO (Å)
ALD Pt30	0.038	57±7	2.73±0.01	3.21±0.04
ALD Pt20Co30	0.030	28±10	2.76±0.02	2.97±0.10

Section S3. Reference

[1] K. Shinozaki, J. W. Zack, R. M. Richards, B. S. Pivovar and S. S. Kocha, *J. Electrochem. Soc.*, **2015**, 162, F1144.

[2] J. Li, Z. Xi, Y. T. Pan, J. S. Spendelow, P. N. Duchesne, D. Su, Q. Li, C. Yu, Z. Yin, B. Shen, Y. S. Kim, P. Zhang and S. Sun, *J. Am. Chem. Soc.*, **2018**, 140, 2926.

[3] M. Brodt, R. Wycisk, N. Dale and P. Pintauro, *J. Electrochem. Soc.*, **2016**, 163, F401.

[4] Q. Jia, K. Caldwell, K. Strickland, J. M. Ziegelbauer, Z. Liu, Z. Yu, D. E. Ramaker and S. Mukerjee, *ACS Catal.*, **2015**, 5, 176.

[5] U. S. DOE, *Fuel Cell Technologies Office Multi-Year Research, Development, and Demonstration Plan*, **2016**.

[6] B. Ravel and M. Newville, *J. Synchrotron Radiat.*, **2005**, 12, 537.
